# What's new about Natsal-3?

**DOI:** 10.1136/sextrans-2013-051292

**Published:** 2013-11-26

**Authors:** Catherine H Mercer, Kaye Wellings, Anne M Johnson

**Affiliations:** 1 Centre for Sexual Health and HIV Research, Research Department of Infection and Population Health, University College London, London, UK; 2 Department of Social and Environmental Health Research, London School of Hygiene & Tropical Medicine, London, UK

**Keywords:** SEXUAL BEHAVIOUR, SEXUAL HEALTH, PUBLIC HEALTH, EPIDEMIOLOGY (GENERAL), HETEROSEXUAL BEHAVIOUR

In November 2013, the initial results from the third National Survey of Sexual Attitudes and Lifestyles (Natsal-3) were published.^1–6^ To coincide with their release, this edition of *Sexually Transmitted Infections* presents two methodological papers^7^ ^8^ explaining the lengthy steps taken to ensure that the data from Britain's latest decennial national probability sample survey of sexual behaviour are reliable and representative of the population. But what are the new challenges and new questions addressed, especially as this is the third such survey?

Studies of sexual behaviour have come a long way since the pioneering but methodologically problematic surveys carried out by Kinsey in the USA in 1948. The emergence of the HIV/AIDS epidemic in the 1980s provided the imperative for the collection of reliable data on sexual behaviour, as epidemiologists and public health specialists recognised the need for accurate estimates with which to predict the likely extent of future spread of HIV in Britain, and the type and scale of prevention activities required. In 1986, plans for a national probability sample survey of sexual attitudes and lifestyles were made. Although feasibility studies had demonstrated the public's acceptance of such a survey and the high quality data obtained,^9^ ^10^ in September 1989, the then prime minister, Margaret Thatcher, vetoed the funding, arguing that it would be an invasion of people's privacy. Subsequently, the Wellcome Trust agreed to fund Natsal-1, and interviews with a probability sample of 18 876 men and women aged 16–59 years from across Britain were completed between May 1990 and November 1991.

The first results from Natsal-1 were published in 1992,^11^ followed in 1994 by detailed analyses in two encyclopaedic books.^12^ ^13^ The data were widely used, not only to model the likely spread of HIV and to plan prevention and treatment services, but to inform public health policy on sex education and contraceptive and other sexual health services. Following the decline in rates of sexually transmitted infection (STI) diagnoses in the 1980s after the emergence of HIV, STI rates began to rise again in the 1990s, and the need for updated behavioural estimates became clear. Natsal-2, funded by the UK Medical Research Council, was undertaken between 1999 and 2002, and interviewed 12 110 people aged 16–44 years. It featured both major methodological developments and a broader range of topics than Natsal-1. The target population was younger, and London residents and ethnic minority populations^14^ were oversampled, as STI/HIV risk was greatest in these populations. While Natsal-1 had used interviewer-administered pen-and-paper questionnaires and self-completion questionnaires for the more sensitive questions, Natsal-2 used computer-assisted personal interviewing (CAPI), including a computer-assisted self-interview (CASI). A methodological experiment had shown that CASI improved the quality and completeness of the data collected without affecting rates of reporting sensitive behaviours,^15^ enabling comparisons to be made between Natsal-1 and Natsal-2. Natsal-2 also included biosampling and collected urine samples, and took advantage of took advantage of the then newly available nucleic acid amplification tests for *Chlamydia trachomatis*^16^ and human papilloma virus (HPV).^17^

The first results of Natsal-2 were published on World AIDS Day 2001, and majored on the increase in the reporting of sexual risk behaviours among men and, in particular, women, and more tolerant sexual attitudes, relative to Natsal-1.^18^ They documented the circumstances of first heterosexual intercourse, including a continued decline in the age at sexual debut,^19^ and presented Britain's first population estimates of *C trachomatis* and factors associated with having the infection.^16^ Since then, over 50 papers have been published from Natsal-2 (see http://www.natsal.ac.uk), influencing and informing sexual and reproductive health policy and practice in Britain, notably the chlamydia screening programme, models for HPV vaccination, and the English government's Teenage Pregnancy Strategy.

The last decade has seen a further shift in the conceptualisation of sexual health,^20^ and so Natsal-3 considers sexual health more broadly. The latest study not only investigates STI/HIV risk, but also aspects of reproductive health, sexual function, non-volitional sex, and the interplay between physical and mental health, and sexual health and well-being throughout the life course. The upper age limit for Natsal-3 was increased to 74 years, and the question topics were expanded. Two psychometrically validated measures—the ‘Natsal-SF’ (a measure of sexual function^21^) and the London Measure of Unplanned Pregnancy^22^—were developed specifically for Natsal-3. Cognitive testing and piloting of new questions with all participants, and questions asked in previous Natsal surveys with older people, were carried out to determine their acceptability and validity. Fieldwork for Natsal-3 began in September 2010, and, 2 years later, interviews with 15 162 people were completed.

STIs remain a key component of the latest study, and urine samples were again collected from a subsample of participants aged 16–44 years in Natsal-3, but this time a range of STIs were tested for, and our initial paper^3^ reports on *C trachomatis*, gonorrhoea, HPV, and HIV antibody (details on the testing procedures are published elsewhere^3^ ^7^).

An important innovation for Natsal-3 was the collection of saliva samples to measure free testosterone with a specially developed assay. This will enable the examination of the relationship between sexual behaviour and physiological, hormonal and lifestyle factors. In addition, the data will be used to establish the normative range of testosterone across the lifespan in men and women—the first time that this has been undertaken in a nationally representative survey. These data are scheduled for publication in 2014.

A further innovation in Natsal-3 has been a qualitative research component, conducted by return visits to survey participants of particular sexual health interest who agreed to participate in a further interview. These rich data will complement the quantitative data collected by the survey, and so improve our understanding of the significance of particular behaviours, and motivations for engaging in them.

In addition to the ‘standard’ Natsal-3 survey, administered as a CAPI with a CASI component, a shorter version of the questionnaire has been run as a web-panel survey. Both the representativeness and the quality of data obtained from web panels are uncertain. The aim of this methodological experiment is to assess whether this is a viable, alternative method of data collection, given the increased access to, and reach of, the web, and the lower cost and quick turnaround of web surveys. As the Office of National Statistics propose administering the UK census online in future, the results of this experiment, due to be published in 2014, are likely to have application beyond future Natsals.

To conclude, Natsal-3 has both matured and moved on. It continues to embrace new technologies for both data collection and biological sampling and to address new areas of research as our conceptualisation of sexual health broadens. The Natsal studies have evolved to ensure that they continue to meet epidemiological and public health needs, and, as such, remain a key resource for informing sexual health policy and practice.
